# In-situ TD-GCMS measurements of oxidative products of monoterpenes at typical vaping temperatures: implications for inhalation exposure to vaping products

**DOI:** 10.1038/s41598-022-14236-4

**Published:** 2022-06-30

**Authors:** Jiping Zhu, Jianjun Niu, Dharani Das, Ashley Cabecinha, Hanan Abramovici

**Affiliations:** 1 Exposure and Biomonitoring Division, Environmental Health Science and Research Bureau, Health Canada, Ottawa, Canada; 2 Office of Cannabis Science and Surveillance, Controlled Substances and Cannabis Branch, Health Canada, Ottawa, Canada

**Keywords:** Environmental sciences, Chemistry

## Abstract

Vaping is gaining in popularity. However, there is still much that remains unknown about the potential risk and harms of vaping. Formation of oxidative products is one of such areas that are not well understood. In this study, we used an in-situ thermal desorption GC/MS method to investigate the formation of oxidative products of several monoterpenes at or below typical vaping temperatures. Among the five tested monoterpenes, the unchanged portion of the parent compound in the vapour varied from 97 to 98% for myrcene to 11–28% for terpinolene. The majority of formed oxidative products in the vapour have a molecular weight of 134 (loss of two hydrogens), 150 (insertion of one oxygen and loss of two hydrogen atoms) or 152 (insertion of one oxygen atom). Three products, likely to be p-(1-propenyl)-toluene, β-pinone and fenchol were also observed. This is the first in-situ thermal desorption GC/MS study to investigate the possible formation of oxidative products of monoterpenes, one of the major components in vaping liquids, at temperatures that are relevant to the vaping process. Although the toxicity of inhaling these oxidative products is not clear yet, allergic and irritation reactions associated with oxidized monoterpene oils are well documented. Therefore, potential adverse effects of inhaling these oxidative products during vaping could be investigated to help support human risk assessment.

## Introduction

Vaping refers to inhalation of vapour from vaping liquids that may contain nicotine or cannabinoids, often with a variety of flavouring ingredients. The vapour is generated by heating, but not combusting a device such as an electronic cigarette (e-cigarette), vape mod, vaporizer or vape pen. Vaping was initially introduced as a smoking replacement therapy and a harm reduction alternative to conventional tobacco cigarette smoking to reduce exposure to cigarette smoke that is known to contain carcinogens and particulate matter, and hence to reduce the detrimental health consequences associated with conventional cigarette smoking^1^.

Since its introduction, vaping has been gaining popularity. The 2017 Canadian Tobacco Alcohol and Drugs Survey reported that 15% (4.6 million) of Canadians aged 15 years and older had ever tried an e-cigarette, an increase from 13% in 2015^2^. Of those who reported having used an e-cigarette in the previous 30 days, 43% reported using a fruit flavour, 22% tobacco flavour and 14% candy/dessert^2^. Vaping is more common among younger people. The 2020 Canadian Tobacco and Nicotine Survey found that about 14% of youth aged 15 to 19 reported having vaped in the past 30 days, and 35% reported having tried it at some point in their lives^3^. The percentages were 13% and 53%, respectively among young adults aged 20 to 24. In comparison, these percentages decreased to 3% and 13% respectively, in adults aged 25 and older^3^.

Vaping liquids contain a complex mixture of chemicals^4^. Besides nicotine or cannabinoids, it also has carrier solvents such as propylene glycol and vegetable glycerin, as well as a variety of flavouring ingredients^5^. When used in foods, these food flavour ingredients with appropriate levels allowed in food have a “generally recognised as safe” (GRAS) status determined by regulatory bodies such as the Flavour and Extract Manufacturers’ Association (FEMA); however, the concentrations of flavouring ingredients in vaping products can be over ten-thousand times higher than in food^1^. It is so far not clear what the health effects may be of inhaling high concentrations of GRAS flavouring ingredients. The potential health impact of inhalation of chemicals, and flavouring ingredients in particular, in vaping liquids has been increasingly recognized as a concern in the past several years^4,6,7^. U.S. Department of Health and Human Services in its Surgeon General report documented a number of studies that detected various flavouring compounds, but not their transformed products, in vaping aerosols some of which may result in adverse health effects when inhaled^8^.

Chemicals in the vaping liquids may produce transformation products in vapor under heat, leading to possible additional toxicities. For example, one recent study has shown that flavouring aldehydes can rapidly react with the carrier solvent in vaping liquids to form aldehyde-propylene glycol acetals that can be inhaled through vaping. These acetals can activate aldehyde-sensitive TRPA1 irritant receptors and aldehyde-insensitive TRPV1 irritant receptors in vitro^9^. Perhaps the most high-profile health impact of vaping was the 2019 outbreak of E-cigarette or vaping product-associated lung injury (EVALI) that sickened thousands and claimed dozens of lives in the U.S. The illness was linked to the use of cannabis vaping products containing Vitamin E acetate, an adulterant on the illegal market^10^. Various mechanisms of action have been postulated to have contributed to or caused the lung damage observed with EVALI including the formation of toxic ketene gas^11^ as well as duroquinone and durohydroquinone^12–14^ from the thermal breakdown of Vitamin E acetate^11^.

Taken together, these findings suggest that vaping may not be without any risk and additional research is needed to better characterize and understand the health risks associated with vaping. So far, most of the studies to characterize vaping aerosols have focused on a limited number of substances including toxic metals, particulate matters, polycyclic aromatic hydrocarbons and aldehydes such as formaldehyde and acetaldehyde^4^ as well as the formation of several small molecules of volatile organic compounds including benzene^15^. Some chemicals in vaping liquids such as saccharides, used to impart a sweet flavour that can appeal to children, can degrade and produce furans and aldehydes when heated^16^.

Among the flavouring ingredients, several monoterpenes were found to be present in nicotine^17^ or cannabis^18^ vaping liquids. Monoterpenes are a group of unsaturated hydrocarbons with a general molecular formula of C_10_H_16_. They are present in essential oils with distinct flavouring characteristics. Despite their GRAS status when used in food and cosmetics, oxidized monoterpenes in light- and air-exposed aged tea tree oil have been shown to cause more allergic reactions when applied to skin compared to freshly made tee tree oil^19^. Degradation products of cannabis-terpene distillate leading to the formation of benzene, methacrolein and isoprene at dabbing and cartridge vaporizers temperatures have also been reported, in which the mechanism of degradation of the monoterpene myrcene was investigated^20^.

Several studies on the thermal degradation of monoterpenes have been reported^21–23^. They included off-line reactions of monoterpenes at 120 °C with oxygen followed by solvent extraction of the reactants for GC/MS analysis^21^, pyrolysis of monoterpenes above 300 °C using pyrolysis-GC/MS^22^ and other methods^23^. So far, However, transformation of monoterpenes in vaping aerosol at relatively low temperature of 100 °C to 200 °C, a temperature range at the lower end of the operating temperature of the vaping devices, using in-situ thermal desorption GC/MS has not yet been reported.

In this study, we investigated the oxidative reactions of several monoterpenes that are used as flavoring ingredients in vaping liquids using an in-situ thermal desorption GC/MS system as a method to gain insight into chemical transformations that may occur during simulated vaping conditions. To achieve this goal, we first trapped the test chemical that had been desorbed from a thermal desorption unit and then heated the chemical at temperatures raging from 100 to 200 °C to deliver the gas phase into a coupled GC/MS for analysis.

## Methods

### Sample preparation

Five monoterpenes, namely α-pinene (purity: ≥ 99.0%), β-pinene (≥ 95.0%), terpinolene (94.0%), myrcene (90.0%) and limonene (≥ 99.0%), were purchased from Sigma-Aldrich Canada (Oakville, Ontario, Canada). Neat chemicals were individually diluted in hexane (dilution ratio: 1:100 to 1:1000). An aliquot (1 to 2 µl) of diluted solution was pipetted onto the glass wool that was placed inside a thermal desorption (TD) tube. TD tubes with the glass wool had been conditioned at 350 °C for 4 h under pure nitrogen gas with a flow rate of 100 ml/min. The prepared tube was inserted to a Thermal Desorption Unit (TDU) from the sampler tray.

### Thermal desorption (TD)

**Figure 1:**
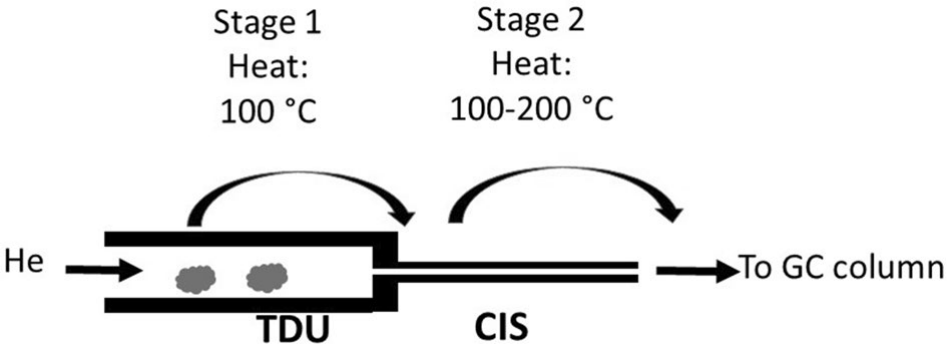
Two-stage thermal desorption process for the study of in-situ oxidative reaction of monoterpenes.

TD system has a two-stage desorption process to deliver the emitted chemicals into the GC column for analysis (Fig. 1). The initial temperature of the thermal desorption unit (TDU) was set at 40 °C. After 0.5 min equilibrium time and 3 min of initial time, the TDU was heated with a ramping rate of 400 °C/min to 100 °C and held at the temperature for 3 min for the desorption of the analyte. The desorbed analyte was trapped at – 10 °C in the cool injection system (CIS), which had an empty quartz liner and was connected downstream of the TDU. A spilt ratio of 1:100 was used for the desorption in TDU, meaning that only 1% of the desorbed material from TDU reached CIS while the rest was vented out. CIS was connected directly to GC column. When the desorption in TDU was completed, the sample tube in TDU was returned to the sampler tray and a clean blank tube was reinserted into the TDU. After an equilibrium time of 0.05 min and an initial time of 3 min at − 10 °C, CIS was heated with a ramping rate of 12 °C/s to a desired temperature of 100 °C, 150 °C and 200 °C, respectively, for three different sample tubes that were spiked with the same compound and kept at the desired temperature for 5 min. CIS was operated in splitless mode. Due to inherited feature of 3 ml/min venting in the thermal desorption system and a selected GC column flow of 1 ml/min, the total CIS desorption flow was estimated at 4 ml/min with 1 ml/min flow entering the GC column. Helium was used as both desorption gas and GC/MS carrier gas.

### GC/MS analysis

Desorbed chemicals from CIS were introduced in-situ to a Gas Chromatograph (GC, Agilent 7890) and Mass spectrometer (MS, Agilent 5977B) for analysis. Chemicals were separated using DB-624 column (60 m long with 0.25 mm I.D., and 1.4 μm film thickness). GC oven temperature program was set as follows: initial temperature: 40 °C (hold for 8 min), ramping: 10 °C/min until 250 °C (hold for 10 min). Temperature for the transfer line connecting to MS was set at 250 °C. MS was operated in positive Electron Impact (EI) mode with an ion source temperature of 230 °C and a quadrupole temperature of 150 °C. The signals were detected in full scan mode with a scan range of 30–350 amu.

### Peak identification

Peaks in the chromatograms were identified by comparing their mass spectrum with that of NIST library, version 17. A match score of greater than 800 (max. 1000) for the spectrum and greater than 50% probability of the suggested chemical structure were used as the threshold to identify possible structures of the peak. In addition, analyst’s knowledge of fragmentation was used to elucidate the structure. Partial structures were proposed if the full structure could not be determined.

## Results

**Figure 2:**
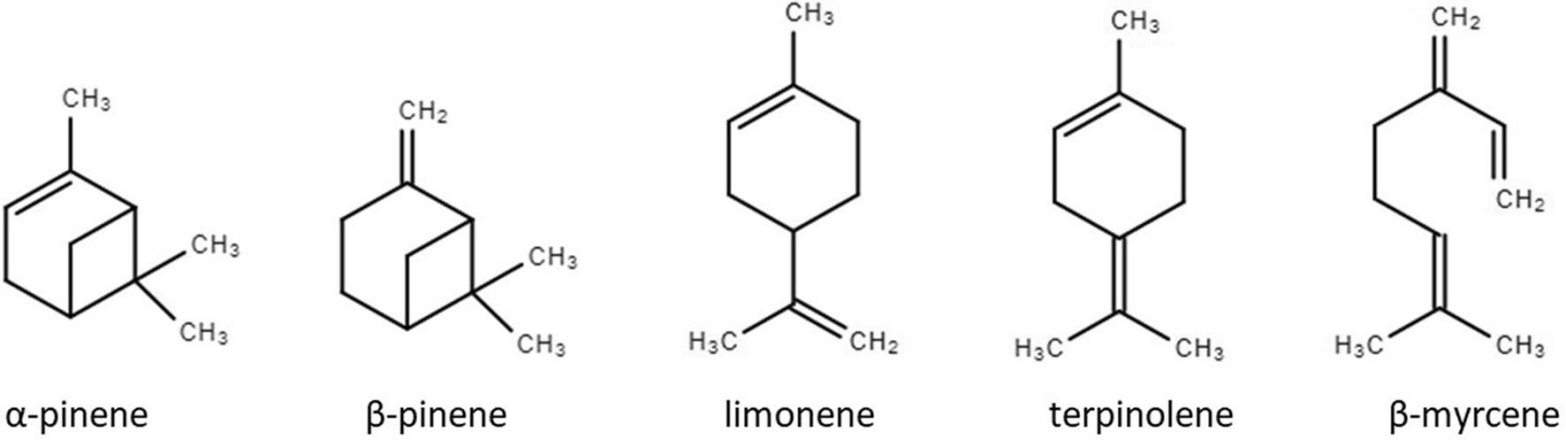
Chemical structure of five monoterpenes investigated in this study.

Five monoterpenes, namely limonene, α-pinene, β-pinene, β-myrcene and terpinolene, have been tested in this study. Structure of these five monoterpenes are depicted in Fig. 2. The purity of these five standards was checked using liquid injection in split mode on another GC/MS instrument (see Supplementary Information, SI). Based on GC/MS total ion chromatogram peak areas, α-pinene, β-pinene and limonene had no noticeable impurities. β-Myrcene standard was 86.15% pure and showed another peak on the chromatogram, which is likely an isomer of β-myrcene. The purity of terpinolene was 90.58%, several small peaks were observed in the chromatogram (Figure S1).

**Figure 3:**
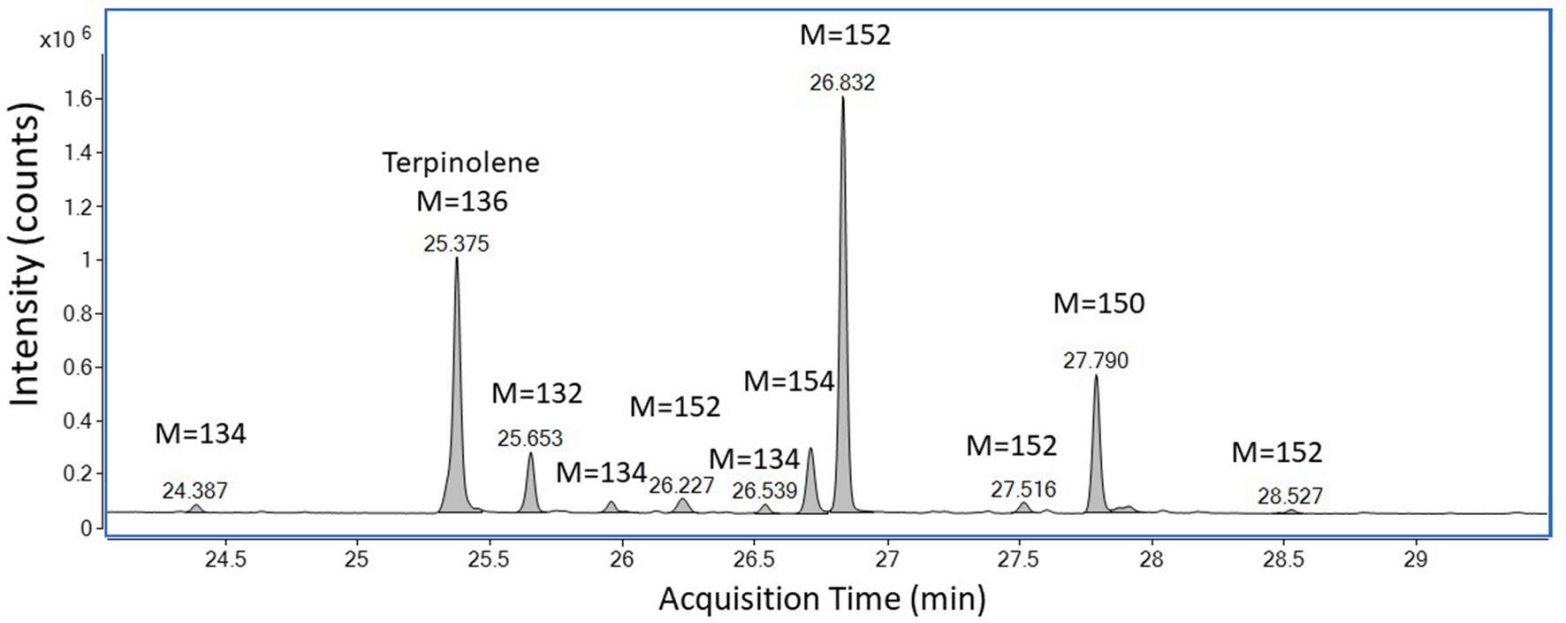
GC/MS chromatogram of terpinolene at TDU of 100 °C and CIS of 150 °C. M: Molecular weight.

A typical total ion chromatogram (TIC) of monoterpenes under the experimental condition is presented in Fig. 3, which shows terpinolene and its oxidative products under the thermal desorption condition. The exact structure of oxidative products was often difficult to confirm, largely due to similarity in the mass spectra among isomers of the structures. As a result, the majority of peaks were identified by their possible molecular weight based on the molecular ion that is recognized in their respective mass spectrum. Detailed information on TIC peak areas of the five monoterpenes at the CIS temperatures of 100 °C, 150 °C and 200 °C are provided in Table S1 to Table S5, respectively, in SI.

Various oxidative products from the five studied monoterpenes, which all have a molecular weight of 136 amu., were identified by their molecular ions (M^+∙^). Identified oxidative products include those with an M^+∙^ of 132 (M-4), 134 (M-2), 138 (M + 2), 150 (M + 14), 152 (M + 16) and 154 (M + 18). Possible oxidative products associated with these molecular ions are suggested in Table 1. For example, a peak with a molecular ion of m/z 134 indicates a loss of two hydrogen atoms [H] and a peak with a molecular ion of m/z 152 indicates an addition of one oxygen atom [O] to the original terpene structure.

**Table 1:** Possible oxidative structures of tested monoterpenes related to observed molecular ions.

Molecular ion	Formula	Change in MW	Oxidative product	Explanation
132	C_10_H_12_	M-4	− 4[H]	Loss of 4 hydrogens to form two double bonds from two –CH_2_–CH_2_− group
134	C_10_H_14_	M-2	− 2[H]	Loss of 2 hydrogens to form an double bond from –CH_2_–CH_2_−
136	C_10_H_16_	M		Parent compound or its isomers
138	C9H_14_O	M + 2	+ [O] and −[CH_2_]	Replacing –CH_2_- group with an oxygen
150	C_10_H_14_O	M + 14	+ [O] and − 2[H]	Formation of C=O group from –CH_2_−
152	C_10_H_16_O	M + 16	+ [O]	Insertion of oxygen between C and H in –CH_2_− CH_2_− group to form hydroxyl group, or addition of oxygen to a double bond to form epoxide group
154	C_10_H_18_O	M + 18	+ [O] and + 2[H]	Addition of OH and H to a double bond

Table 2 summarizes the percentage of oxidative products from each of the five monoterpenes. The products were grouped by their molecular ions identified in the desorbed gas phase under three different CIS desorption temperatures along with the number of peaks identified in each group.

**Table 2:** Percentage of total ion chromatogram (TIC) peak areas and number of peaks for each molecular ions observed in the TD-GC/MS analysis of monoterpenes.

	CIS Temperature	Parent (M = 136)	M = 132^	M = 134	Other M = 136	M = 138^	M = 150	M = 152	M = 154^
α-Pinene	100 C	42.6	ND	10.0	12.7	ND	3.7	31.0	ND
	150 C	78.7	ND	4.1	5.9	ND	1.9	9.3	ND
	200 C	60.4	ND	9.4	9.4	ND	4.5	16.3	ND
	Average	60.6	NA	7.8	9.3	NA	3.4	18.9	NA
	# of Peaks	NA	NA	(5)	(2)	NA	(2)	(4)	NA
β-Pinene	100 C	74.1	ND	1.6	7.3	3.1	4.8	9.0	ND
	150 C	73.4	ND	0.4	9.9	2.4	5.0	8.8	ND
	200 C	52.4	ND	1.0	11.7	6.6	9.4	18.9	ND
	Average	66.7	NA	1.0	9.6	4.0	6.4	12.3	NA
	# of Peaks	NA	NA	(2)	(5)	(1)	(2)	(3)	NA
Myrcene	100 C	97.5	ND	ND	2.5	ND	ND	ND	ND
	150 C	98.0	ND	0.0	1.9	ND	ND	ND	ND
	200 C	97.8	ND	0.2	2.1	ND	ND	ND	ND
	Average	97.8	NA	0.1	2.2	NA	NA	NA	NA
	# of Peaks	NA	NA	(1)	(3)	NA	NA	NA	NA
Limonene	100 C	83.9	ND	1.1	ND	ND	3.8	11.1	ND
	150 C	93.3	0.2	1.2	0.2	ND	1.8	3.4	ND
	200 C	92.0	0.4	1.5	0.1	ND	2.3	3.7	ND
	Average	89.7	0.3	1.3	0.2	NA	2.6	6.1	NA
	# of Peaks	NA	(1)	(2)	(1)	NA	(2)	(9)	NA
Terpinolene	100 C	13.1	3.7	3.3	ND	ND	14.3	55.3	8.8
	150 C	27.6	6.1	3.1	ND	ND	13.3	41.5	7.2
	200 C	11.4	23.9	6.5	ND	ND	35.8	14.1	7.9
	Average	17.4	11.2	4.3	NA	NA	21.1	37.0	7.9
	# of Peaks	NA	(1)	(4)	NA	NA	(1)	(5)	(1)

ND, not detected; NA, not applicable.

*System residual is the sum of peak areas detected in the blank, presented as percentage of sum of peaks area in the sample run.

^Possible structure: M = 132, p-(1-propenyl)-toluene (C_10_H_12_); M = 138, β-pinone (C_9_H_14_O); M = 154, fenchol (C_10_H_18_O). Mass spectra of these three peaks are provided in Figure S2, S3 and S4, respectively, in SI.

The percentage of unreacted parent compounds that was present in the desorbed gas phase was also included in Table 2. The amount of parent compound and its oxidative products was estimated from the TIC peak areas assuming all compounds have the sample response in TIC. The unchanged portion of parent monoterpenes in the gas phase of the thermal desorption was in the order of myrcene (97–98%) > limonene (81–92%) > α-pinene (60–80%) > β-pinene (50–73%) > terpinolene (11–28%), indicating differing thermal stabilities among the studied terpenes against oxidation at temperature range of 100 °C to 200 °C. Due to low desorption temperatures (100 °C in TDU and 100 °C to 200 °C in CIS) used in this study, evaporation of monoterpenes may not be complete resulting in possible variation in the amount of parent chemical and its oxidative products being introduced in-situ into the GC/MS, therefore the relative concentrations presented in Table 2 (portions of parent compound relative to formed transformation oxidative products) should be interpreted with caution and should not be considered quantitative results. However, these findings do indicate the reactive nature of the monoterpenes observed under the experimental conditions of this study.

Besides parent monoterpenes, several peaks with a molecular ion of 136 were also observed (Table 2). These peaks are likely a result of thermal rearrangement of the parent monoterpenes^23^. The highest number of additional peaks of M = 136 was from the reaction of β-pinene (5 peaks), followed by myrcene (3 peaks), α-pinene (2 peaks), limonene (1 peak). Terpinolene did not produce additional M = 136 products. Common oxidative products of monoterpenes are those with molecular ion of M = 134, M = 150 and M = 154. M = 134 peaks were observed in the reaction of all five monoterpenes, but they were most abundant in the reaction of α-pinene (7.8%, 5 products) and terpinolene (4.3%, 4 products) (Table 2). These are likely formed by loss of two hydrogen atoms resulting in the formation of a new carbon–carbon double bond or new aliphatic ring (Table 1). Oxidative products M = 150 and M = 152 were also present in the reaction of monoterpenes, except for myrcene. Yields of these two groups of products were highest in the reaction of terpinolene with 21.1% and 37.0% for M = 150 and M = 152, respectively. That was followed by α-pinene, β-pinene and limonene in decreasing order.

Possible structures of three oxidative products were tentatively identified (Table 2). Oxidative product with the molecular ion of 132 is likely to be p-(1-propenyl)-toluene (Table 2), which was observed only in the reaction of terpinolene (11.2%) and limonene (0.3%). Oxidative product with the molecular ion of 138 is likely to be β-pinone (C_9_H_14_O), which was only observed in the reaction of β-pinene (4.0%). Oxidative product with the molecular ion of 154 is likely to be Fenchol (C_10_H_18_O), which was only observed in the reaction of terpinolene (7.9%). Fenchol was also detected in 6 liquids, 7 vapours and 9 aerosols, respectively, among 12 cannabis vapour oil cartridge samples collected in California, USA^24^. Mass spectra of these three peaks are provided in Figures S2, S3, and S4 respectively, in the SI.

## Discussion

We investigated the reaction products of five monoterpenes in the gas phase when these flavouring chemicals are heated inside a thermal desorption (TD) system described in the previous section. We selected a temperature of 100 °C in the TDU (first stage, Scheme 1) to allow the evaporation of the testing compounds, which have a boiling point range of 150 – 200 °C, and transferring them to CIS. The relatively low temperature in TDU was selected to minimize possible reactions of the compound during the first stage desorption. Temperature effects on the reactions of monoterpenes were then studied during the second stage desorption (Scheme 1) in CIS with three temperatures of 100 °C, 150 °C and 200 °C, respectively. Most of the vaping devices have a heating zone operated at the temperature range that provide sufficient heat to evaporate the vaping components while avoiding burning. Common vaping temperatures in vaping devices are in the range of 100 °C to 300 °C^25^. A lower temperature range of 100 °C to 200 °C allows us to demonstrate that even at the lower end of the typical vaping operating temperatures, monoterpenes still can undergo thermal oxidation to form various products. Higher vaping temperatures will likely result in more monoterpenes being oxidized to form oxidative products.

Oxidative reactions of monoterpenes in the gas phase are initiated by ozone, hydroxyl radicals, and nitrate (NO_3_) radicals, as well as UV light. Ozonolysis of monoterpenes occurs mainly through electrophilic attack of the carbon–carbon double bond by ozone^26^. Gas phase oxidative reactions of monoterpenes in the presence of ozone has been demonstrated through chamber studies^27^. The reactions of monoterpenes under the TD operating conditions in this study were likely initiated by the trace amount of ozone present in the lab air that enters the TD system during thermal desorption, as ozone is ubiquitously present in both indoor air and outdoor air^28^. Mass spectrometer in the TD-GC/MS system is operated under vacuum. As a result, a small amount of air is sucked into the TD system from the surrounding air during changing and loading of sample tubes and possible leakage in various seals used in the thermal desorption system. The presence of air in the sample runs was evident by the detected background levels of m/z 32 in the spectrum. In the real-world context of vaping, the amount of air present in the heating block of a vaping device would be much more prevalent as the air is drawn directly through the heating block of the vaping device when it is heated for inhalation. As a result, thermal oxidative reactions could be even more prevalent in the real vaping operation compared to the results presented in this paper.

Thermal degradation pathways of monoterpenes have been recently reviewed^23^. One of the pathways is the oxidative degradation, which includes dehydrogenation to form products with M = 134 and M = 132, epoxidation to form products with M = 150, C=C double bond oxidative cleavage to form products with M = 138, and allylic oxidation to form products with M = 150 (ketone) and M = 152 (alcohol). Various oxidative products observed in our study (M = 132, M = 134, M = 150 and M = 154) agree with these reported oxidative degradation pathways^21,23^.

Monoterpenes are considered GRAS in foods and other products^29^. However, monoterpenes are sensitive to heat and light and can degrade under those conditions over time, and therefore protecting them from oxidation is very important to maintain their quality/integrity as well as for their safe use^30^. For example, one study investigated the allergic properties of aged tea tree oil that had been exposed to air and light, from a few days to several months, and found that the aged oil was more viscous and when applied to skin it caused more allergic reactions^19^. A UK study tested 4731 patients and found 411 (8.7%) patients had positive patch test reactions to limonene hydroperoxide or linaool hydroperoxide or both. Of the 3297 patients who recorded irritant reactions, there were 242 irritant reactions to one or both limonene and linalool hydroperoxides, compared to only 15 irritant reactions to the non-oxidized terpenes^31^.

The initial study on the human health impact of inhalation of monoterpenes and their oxidation products was a result of investigation of health complaints including throat irritation in buildings^32^. The sensory and pulmonary health impacts of inhalation exposure to oxidation products of monoterpenes have been recorded through epidemiology studies, in-vitro/in vivo toxicology studies and controlled human exposure studies. Increased sensory and pulmonary irritation due to exposure to oxidative products of monoterpenes are evident, especially at high concentrations^33,34^. Although in most indoor settings with low ozone and monoterpene levels, ozone-initiated monoterpene reaction products may not be a significant health concern^35^, levels of monoterpenes and their oxidative products in the vapor during vaping might be sufficiently high to cause adverse health effects such as airway irritation observed in in vivo studies^36^.

Real-life examples of vaping aerosol exposures are complex to simulate in the laboratory settings. Our study demonstrated an in-situ generation and analysis system using thermal desorption as a surrogate method for vaping. Although this in-situ thermal desorption is different from real-world vaping where the liquid is being heated and cooled repeatedly over a series of puffs. Nevertheless, oxidative reactions of monoterpenes that are observed under heat in the common vaping temperature range may be indicative of possible chemical reactions that could occur during vaping. Therefore, this study design could be useful as it offers potential targeted transformation candidates to further study aerosol transformation and user exposures. Through the example of monoterpenes in this study we also raise the awareness that many thermally liable chemicals flavoring ingredients in vaping liquids or solids may be undergoing similar oxidative reactions and their transformed products will be inhaled that may have health consequences that we currently have no sufficient knowledge of.

## Supplementary Information

Supplementary Information.

## Data Availability

Original data including mass spectrum will be available upon request to corresponding author.
