# Supplementary material for: In-situ TD-GCMS measurements of oxidative products of monoterpenes at typical vaping temperatures: implications for inhalation exposure to vaping products
Source: Sci Rep. 2022 Jun 30;12:11019. doi: 10.1038/s41598-022-14236-4 (PMC9247066; doi:10.1038/s41598-022-14236-4)
Supplement: Supplementary file 1 — Supplementary Information. [file 41598_2022_14236_MOESM1_ESM.pdf]

Supplementary Information

In-situ TD-GCMS measurements of oxidative products of monoterpenes at typical vaping temperatures: implications for inhalation exposure to vaping products

Jiping Zhu[1,*], Jianjun Niu[1], Dharani Das[1], Ashley Cabecinha[2] and Hanan Abramovici[2]

1: Exposure and Biomonitoring Division, Environmental Health Science and Research Bureau, Health Canada, Ottawa, Canada

2: Office of Cannabis Science and Surveillance, Controlled Substances and Cannabis Branch, Health Canada, Ottawa, Canada

The support information (SI) contains five tables and four figures.

Section 1: Peak areas and suggestive identification of GC/MS chromatograms of five monoterpenes

Table S1. Peak areas and suggestive Identification of peaks in total ion chromatogram (TIC) of α-pinene and its oxidative products.

100 C			150 C			200 C				
RT	Area	%Area	RT	Area	%Area	RT	Area	%Area	MW	Compound
22.24	149803	11.30	22.24	1010403	27.70	22.25	331683	13.10	136	α-pinene
22.49	412871	31.30	22.49	1864297	51.00	22.49	1200936	47.30	136	α-pinene
22.87	39374	3.00		ND	NA	22.87	37826	1.50	134	
22.95	149652	11.30	22.96	164720	4.50	22.96	175940	6.90	136	
23.05	54661	4.10	23.06	99579	2.70	23.06	134296	5.30	134	
23.9	10536	0.80		ND	NA		ND	NA	134	
24.32	17824	1.30	24.33	51701	1.40	24.33	62194	2.50	136	
24.39	22389	1.70		ND	NA	24.39	36161	1.40	134	
26.03	41546	3.10	26.02	24683	0.70	26.02	31269	1.20	152	
26.54	4502	0.30	26.55	50404	1.40	26.55	30199	1.20	134	
26.84	257008	19.50	26.84	164544	4.50	26.84	199677	7.90	152	
27.19	40500	3.10	27.20	79375	2.20	27.2	110811	4.40	152	
27.61	70826	5.40	27.61	71721	2.00	27.62	71761	2.80	152	
27.67	22264	1.70	27.66	23855	0.70	27.67	48197	1.90	150	
28.28	26659	2.00	28.28	46946	1.30	28.28	66134	2.60	150	
Total	1320414	100.0		3652227	100.0		2537084	100.0		

ND = Not Detected; NA = Not Applicable.

Table S2. Peak areas and suggestive Identification of peaks in total ion chromatogram (TIC) of β-pinene and its oxidative products.

100 C			150 C			200 C				
RT	Area	%Area	RT	Area	%Area	RT	Area	%Area	MW	Compound
22.50	63515	3.04	22.49	141724	4	22.49	70058	4.42	136	
	ND	NA	22.88	12281	0.35	22.88	5743	0.36	136	
22.96	36344	1.74	22.96	133920	3.78	22.95	71861	4.53	136	
23.25	33049	1.58	23.05	2397	0.07	23.06	5946	0.38	134	
23.36	943959	45.23	23.36	1628143	45.96	23.37	287983	18.17	136	β-pinene
23.52	603300	28.91	23.52	973211	27.47	23.52	542558	34.23	136	β-pinene
24.27	22455	1.08		ND	NA		ND	NA	136	
24.33	30821	1.48	24.33	61474	1.74	24.33	37482	2.36	136	
	ND	NA	24.39	12886	0.36	24.39	10629	0.67	134	
27.19	85215	4.08	27.19	161189	4.55	27.19	140452	8.86	152	
27.50	65384	3.13	27.49	85996	2.43	27.50	104202	6.57	138	β-pinone
27.66	40728	1.95	27.67	89477	2.53	27.67	73207	4.62	150	
28.01	54380	2.61	28.02	89863	2.54	28.01	66127	4.17	152	
28.10	48702	2.33	28.11	61680	1.74	28.11	93628	5.91	152	
28.28	59048	2.83	28.28	88559	2.5	28.28	75076	4.74	150	
Total	2086900	100.0		3542802	100.0		1584952	100		

ND = Not Detected; NA = Not Applicable.

Table S3. Peak areas and suggestive Identification of peaks in total ion chromatogram (TIC) of β-myrcene and its oxidative products.

100 C			150 C			200 C				
RT	Area	%Area	RT	Area	%Area	RT	Area	%Area	MW	Compound
23.22	1716916	38.63	23.22	2413531	17.41	23.22	2239783	12.97	136	β-myrcene
23.33	2618789	58.91	23.33	11176576	80.63	23.33	14638427	84.79	136	β-myrcene
23.82	49162	1.11	23.82	58789	0.42	23.83	61599	0.36	136	
23.94	60210	1.35	23.94	187861	1.36	23.94	249857	1.45	136	
	ND	NA	24.33	20389	0.15	24.33	45763	0.27	136	
	ND	NA	24.40	4862	0.04	24.40	29466	0.17	134	
Total	4445077	100.0		13862009	100.0		17264895	100.0		

ND = Not Detected; NA = Not Applicable.

Table S4. Peak areas and suggestive Identification of peaks in total ion chromatogram (TIC) of limonene and its oxidative products.

100 C			150 C			200 C				
RT	Area	%Area	RT	Area	%Area	RT	Area	%Area	MW	Compound
			23.91	25575	0.23	23.91	22267	0.14	134	
24.25	807215	38.29	24.25	1923209	17.12	24.25	1758653	10.72	136	limonene
24.33	961985	45.64	24.33	8553513	76.15	24.33	13330596	81.24	136	limonene
24.38	24116	1.14	24.39	112376	1.00	24.39	222962	1.36	134	
	ND	NA	25.36	19518	0.17	25.36	22572	0.14	136	
	ND	NA	25.66	21225	0.19	25.65	65346	0.40	132	p-(1-propenyl)-toluene
26.63	24637	1.17	26.63	29378	0.26	26.63	75274	0.46	152	
26.7	78706	3.73	26.7	27716	0.25	26.7	48620	0.30	152	
26.76	45582	2.16	26.75	23799	0.21	26.76	41502	0.25	152	
26.91	16529	0.78	26.9	23017	0.20	26.9	74494	0.45	152	
27.52	17513	0.83	27.52	29440	0.26	27.52	43450	0.26	152	
27.77	16108	0.76	27.78	14551	0.13	27.79	20113	0.12	150	
	ND	NA	27.99	11967	0.11	27.98	29698	0.18	152	
	ND	NA	28.14	23064	0.21	28.14	17762	0.11	152	
28.28	33303	1.58	28.28	123630	1.10	28.28	147762	0.90	152	
28.48	18379	0.87	28.49	86174	0.77	28.49	124510	0.76	152	
28.88	63830	3.03	28.88	184089	1.64	28.88	364204	2.22	150	
Total	2107903	100		11232241	100		16409784	100		

ND = Not Detected; NA = Not Applicable.

Table S5. Peak areas and suggestive Identification of peaks in total ion chromatogram (TIC) of terpinolene and its oxidative products.

100 C			150 C			200 C				
RT	Area	%Area	RT	Area	%Area	RT	Area	%Area	MW	Compound
24.39	38573	0.67	24.39	64814	0.85	24.39	51300	1.15	134	
25.38	760285	13.29	25.38	2129840	27.92	25.38	508802	11.41	136	terpinolene
25.65	216137	3.78	25.65	469306	6.15	25.65	1067235	23.93	132	p-(1-propenyl)-toluene
	ND	NA		ND	NA	25.78	18891	0.42	?	
25.96	88675	1.55	25.96	107128	1.40	25.96	118490	2.66	134	
26.22	104357	1.82	26.23	153823	2.02	26.23	79088	1.77	152	
26.54	67108	1.17	26.54	69492	0.91	26.54	72418	1.62	134	
26.71	509180	8.9	26.71	555264	7.28	26.71	350482	7.86	154	fenchol
26.83	3004544	52.51	26.83	2914671	38.21	26.84	385986	8.65	152	
27.51	101583	1.78	27.52	100491	1.32	27.52	61906	1.39	152	
27.79	831183	14.53	27.79	1029460	13.50	27.79	1596167	35.79	150	
	ND	NA		ND	NA	27.87	103839	2.33	152	
	ND	NA		ND	NA	28.04	45529	1.02	134	
	ND	NA	28.53	33789	0.44		ND	NA	152	
Total	5721624	100.0		7628078	100.0		4460133	100.0		

ND = Not Detected; NA = Not Applicable.

Section 2: **Experimental conditions for checking purities of purchased standards of the five monoterpenes**

The analysis for checking the purities of the standards were carried out using an Agilent 7890 GC/7200 QTOF GC/MS (gas chromatograph/mass spectrometer) (Agilent Technologies, CA, USA) and fitted with a CTC-PAL Multi-Purpose autosampler (Agilent Technologies, CA, USA). Electron impact (EI, at 70 eV) ionization mode was used. for MS operation. Temperature of the source and the quadrupole was set at 230 °C and 150 °C, respectively. The MS was operated in scan mode with a range of 30 amu. to 600 amu. All terpene standards and terpene blends were diluted using hexane. The dilution factors were 50 or 100 times to achieve the proper intensity of peaks in the GC/MS chromatogram. Diluted standard solutions were injected through liquid injection port in split mode (ratio: 100:1). A DB-624 UI column (30 m in length 0.25 mm in diameter 0.25 µm of film thickness, Agilent Technologies, CA, USA) was utilized for the separation of peaks. The oven temperature was programmed as: initial temperature of 40 °C for 5 min, increased at 10 °C/min to 250 °C and kept at that temperature for 10 min. Temperature of the injector port and transfer line was maintained at 230 °C and 260 °C, respectively. Flow of helium gas used as the carrier gas was set 1.3 mL/min.

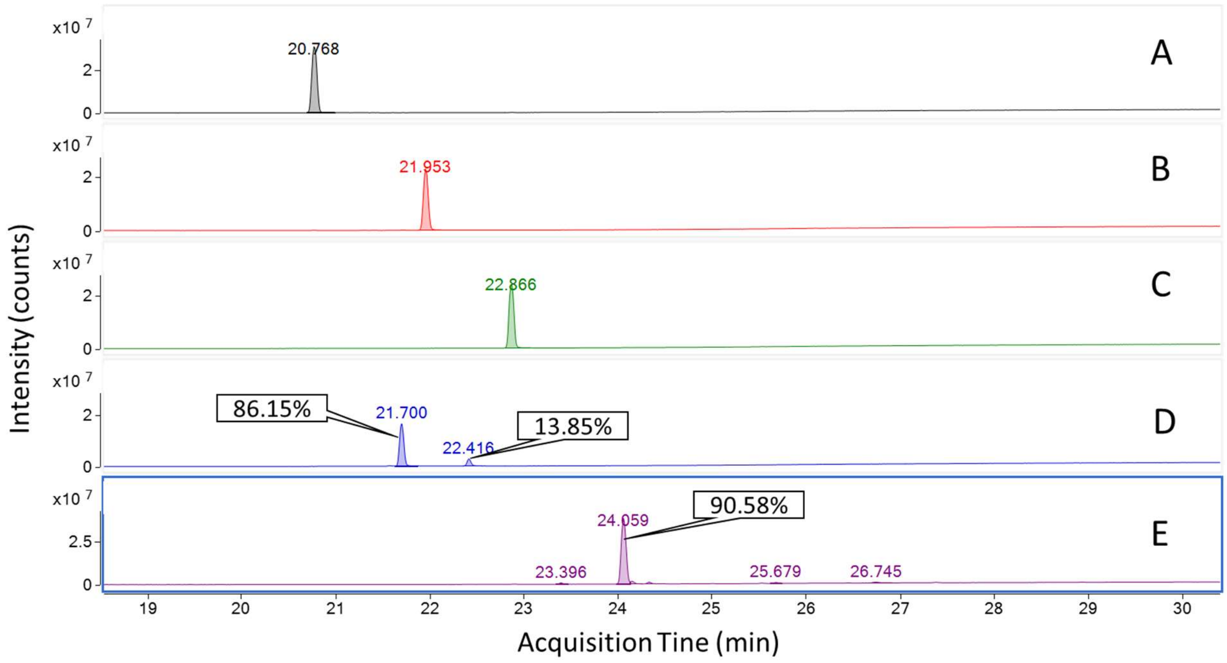

Figure S1: GC/MS total ion chromtograms of five monoterpene standards. A: α-pinene; B: β-pinene; C: limonene; D: β-myrcene; and E: terpinolene. The purity of β-myrcene expressed as percentage of peak area (chromatogram D) was 86.15% (at 21.700 min) with another major peak at 22.416 min that has a very similar mass spectrum and is likely to be α-myrcene. The purity of terpinolene expressed as percentage of peak area (chromatogram E) was determined to be 90.58% (peak at 24.059 min). The purity for the other three standards was determined to be near 100% as there was no other noticable peaks present in the chromatiograms (A, B and C).

Section 3. **Mass spectra of three peaks as oxidative products of monoterpenes in the GC/MS chromatograms That are rentatively identified based on their matching spectrum in the NIST MS Library.**

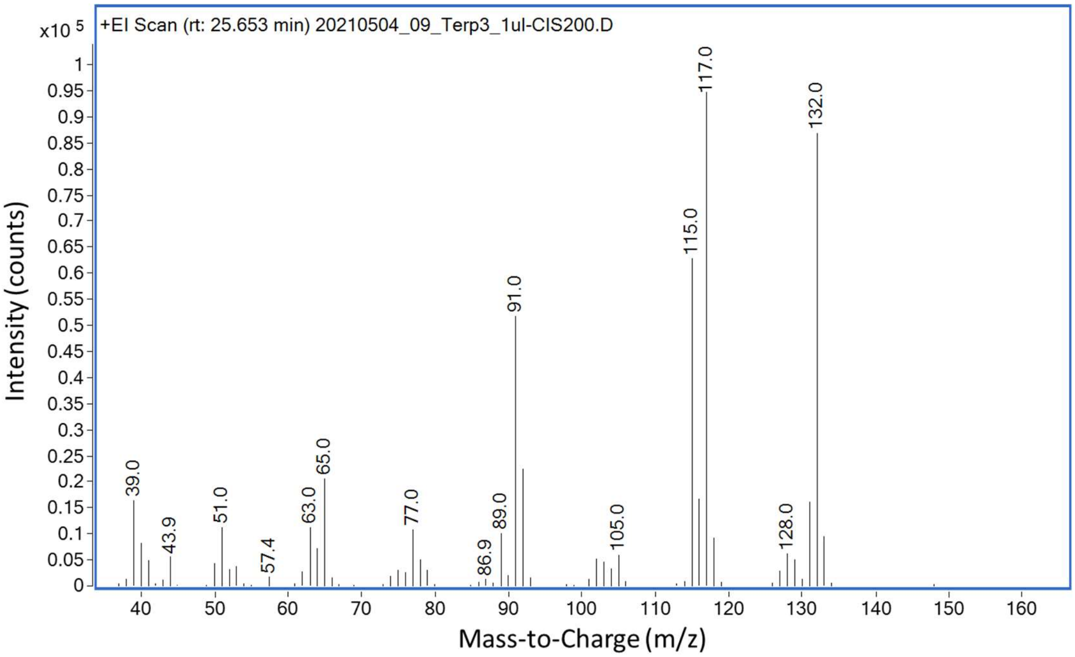

Figure S2: Mass spectrum of GC/MS chromatogram peak at 25.65 min, tentatively identified as p-(1-propenyl)-toluene.

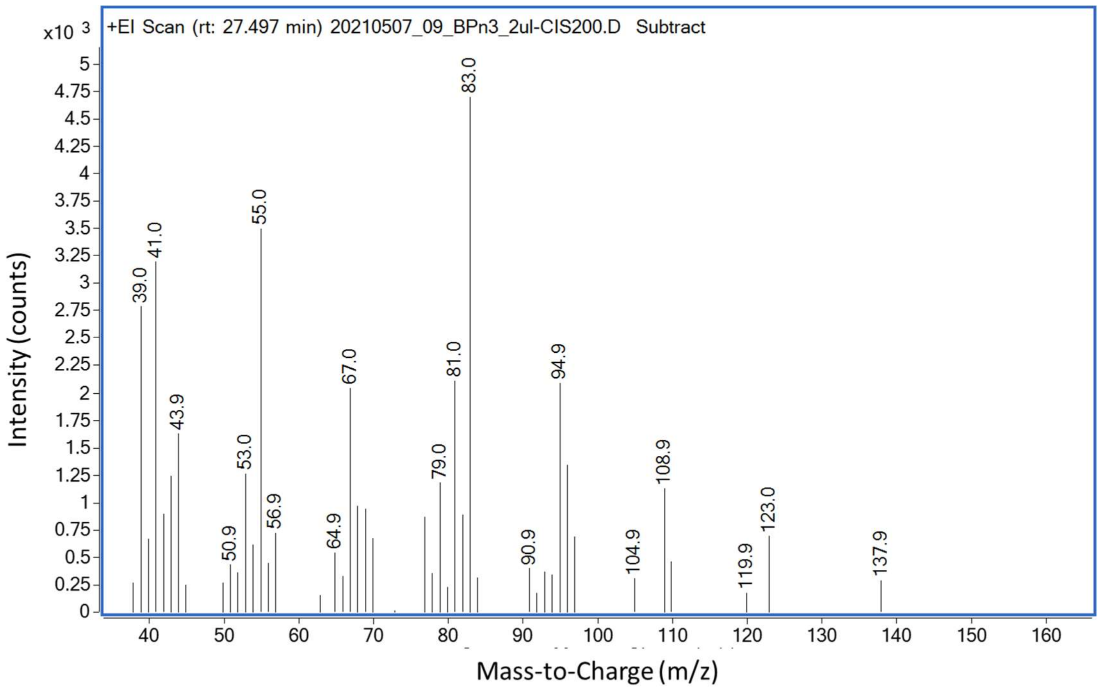

Figure S3: Mass spectrum of GC/MS chromatogram peak at 25.65 min, tentatively identified as β-pinone.

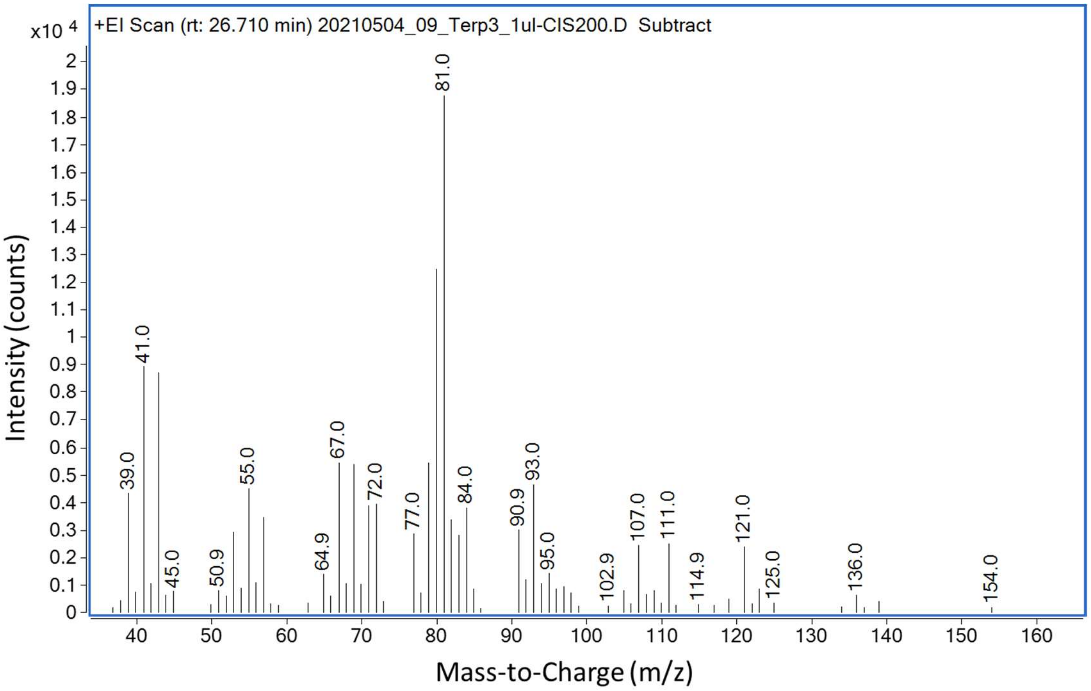

Figure S4: Mass spectrum of GC/MS chromatogram peak at 25.65 min, tentatively identified as fenchol.
